# Sedentary behaviour and level of physical activity among people with post COVID-19 condition: associated factors and changes over time

**DOI:** 10.1186/s13104-025-07177-4

**Published:** 2025-03-20

**Authors:** Elin Östlind, Åsa B Tornberg, Elisabeth Ekstrand, Iben Axén, Christina Brogårdh, Agneta Malmgren Fänge, Kjerstin Stigmar, Eva Ekvall Hansson

**Affiliations:** 1 Department of Health Sciences, Lund University, Lund, Sweden; 2 Dalby Primary Healthcare Centre Region Skåne, Dalby, Sweden; 3 Department of Hand Surgery, Skåne University Hospital, Malmö, Sweden; 4 Institute of Environmental Medicine, Karolinska Institute, Stockholm, Sweden; 5 Department of Neurology, Rehabilitation Medicine, Geriatrics and Memory Disorders, Skåne University Hospital, Lund, Sweden; 6 Ear-Nose and Throat Department, Skåne University Hospital, Lund, Sweden

## Abstract

**Objective:**

To explore the associations between daily time in sedentary behaviour (SED) and physical activity (PA) with perceived physical capacity, health-related quality of life (HRQoL) and fatigue in persons with post COVID-19 condition (PCC). An additional objective was to describe changes in SED and PA levels over three months.

**Materials and methods:**

Individuals with PCC self-reported on physical capacity, HRQoL and fatigue. Accelerometers were used to assess daily SED and light (L), or moderate/vigorous (MV) PA, and data were collected during seven consecutive days at two assessments occasions three months apart. Spearman’s rho and Wilcoxon signed rank test were applied in data analyses.

**Results:**

Fifteen individuals with PCC were included, all women with a mean age of 48 years. A significant positive correlation (r_s_ =0.65) was found between perceived physical capacity and time spent in MVPA. A significant negative correlation (r_s_ = − 0.56) was found between fatigue and time spent in MVPA. No correlations were found for SED, LPA and HRQoL. There were no significant differences in SED and PA levels over time.

**Conclusions:**

We found that good perceived physical capacity and low self-reported fatigue were moderately associated with more daily time in MVPA in persons with PCC. Hence, it is important to evaluate physical activity and fatigue when recommending PA among people with PCC.

**Supplementary Information:**

The online version contains supplementary material available at 10.1186/s13104-025-07177-4.

## Introduction

Recent research has shown that insufficient physical activity (PA) is one of the major risk factors for severe outcomes of COVID-19 [1]. Despite indications that exercise-based rehabilitation is important in treatment of post COVID-19 condition (PCC) [2], there is limited knowledge regarding PA levels among individuals with this condition. When investigating the effects of interventions on common outcomes such as PA level, fatigue, and physical capacity, it is relevant to identify PA levels over time and to be aware of any associations between PA and common consequences of PCC.

The objective of this study was to explore the associations between daily time spent in sedentary behaviour (SED) and PA levels with perceived physical capacity, health related quality of life and fatigue in persons with PCC. An additional objective was to describe changes in SED and PA levels after three months. For this study, a sub-sample of persons responding to a follow-up survey for a larger project (Life After Covid, LAC) investigating how PCC influence everyday life and perceived health was used. Baseline data in the LAC project were obtained through an online survey active from October 2021 to February 2022. These data have been published elsewhere [3, 4, 5, 6, 7]. A follow-up survey was posted in June 2022. Participants who responded to the follow-up online survey and accepted to wear an accelerometer to measure PA were included in the present study.

## Methods and materials

### Study design

This study applied a cross-sectional design, where self-reported physical capacity, HRQoL and fatigue were assessed. In addition, daily SED and level of PA were objectively measured twice, three months apart. The manuscript is written in accordance with the STROBE checklist.

## Recruitment and participants

The inclusion criteria for participation in this study were: having no severe diseases such as fibromyalgia, heart condition or chronic fatigue syndrome, and having symptoms consistent with PCC for two to eight months. No other inclusion or exclusion criteria were used. The recruitment process is illustrated in figure 1 in supplementary material.

Caption figure 1 (in supplementary material).

Figure 1. Flowchart of the recruitment process

### Outcomes

*SED and PA* were assessed as average daily minutes in SED, low physical activity (LPA) and moderate to vigorous physical activity (MVPA), by use of a waist-worn accelerometer, ActiGraph wGT3X-BT (ActiGraph, Pensacola, FL, USA) which has shown fair to moderate correlations with doubly labelled water in assessing PA energy expenditure [8].

*Self-reported physical capacity* was assessed through the Rating of Perceived Capacity (RPC) scale [9]. The RPC is based on metabolic equivalents (METs) that are linked to physical activities on a progressive scale. The most strenuous activity that can be sustained for at least 30 min is rated from 1 (sit) to 20 (elite aerobic training). Self-reported *health-related quality of life* (HRQoL) was measured by the EuroQol visual analogue scale (EQ VAS) [10]. The EQ VAS measures overall health and consists of a vertical scale ranging from 0 to 100 were 0 is the *worst imaginable health* and 100 is the *best imaginable health* [11]. Self-reported *fatigue* was measured by the Fatigue Severity Scale (FSS). The FSS is a self-reported scale that measures the severity and impact of fatigue [12], and consists of 9 statements about the impact of fatigue in daily life. The participants indicate the extent to which they agree or disagree with statements ranging from 1 (strongly disagree) to 7 (strongly agree). The total score is the mean value of the statements and ranges from 1 to 7. A higher score indicates greater fatigue severity and a score exceeding 4 is often used as a cut-off indicating more problematic fatigue [12].

*Sociodemographic characteristics* such as postsecondary education and symptoms related to PCC were collected to describe the sample. Information about age and sex were collected when distributing the questionnaire in fall/winter 2021–2022 (baseline in the LAC-project).

### Data collection

Data on SED and PA were collected during seven consecutive days at two occasions three months apart (June/July and September/October 2022). The accelerometers were mailed to the participants together with a waist-strap and information that it should be worn at the right hip. They were asked to start wearing the accelerometer at a predetermined date, during waking hours. It had to be removed if the participants were in contact with water (showering/swimming etc.). Figure 2 in the supplementary material presents a timeline of the data collection.

Caption figure 2 (in the supplementary material).

Figure 2. Data collection timeline.

The self-reported data were collected with a secure, web-based application, Research Electronic Data Capture (REDCap) [13], through a six-month follow-up questionnaire in the LAC-project. A link to the follow-up online questionnaire was sent to the participants’ e-mail in June 2022.

### Data analysis

Participants who did not respond to the follow-up survey, lacked PA-data for the first measurement period, or no longer had PCC symptoms at the follow-up were excluded from the analyses. Only participants with accelerometer data from both measurement occasions were included in the analyses. The accelerometer data were analysed with ActiLife 6.0 (Actigraph, Pensacola, FL, USA) applying a sampling rate of 30 hz and epoch length at 60 s. Non-wear time was excluded from the analysis and was set to 60 consecutive minutes of no activity registered. Freedson Adult (1998) cut point values for SED, LPA and MVPA were applied; sedentary: 0–99 counts per minute (cpm), LPA: 100–1951 cpm and MVPA over1952 cpm [14]. A minimal wear-time of four days and 600 min per day is recommended [15]. However, due to the few participants with valid data in our study, we conducted a descriptive sensitivity analysis with different minimal wear-times to ensure that participants were not unnecessarily excluded. A minimal wear-time of three days and 500 min rendered similar results as four days and 600 min (Table 1 in the supplementary material). The central tendency of daily SED, LPA and MVPA time were calculated for the two measurements separately. Due to non-normal distributions, data are presented as median and interquartile range (IQR). Wilcoxon signed-rank test was used to compare SED and PA data from the two measurements.

Participants’ sociodemographic characteristics are presented as mean, standard deviation (SD) or proportions (%). The self-reported variables (physical capacity, HRQoL and fatigue) are presented as median and IQR due to non-normal distributions. Spearman’s rank correlation was used to calculate the associations between SED, LPA, MVPA data from measurement occasion 1 and the self-reported outcomes (physical capacity, HRQoL, fatigue). The strength of the correlations was interpreted as: rho < 0.5 low, 0.5 to < 0.7 moderate, > 0.7 high [16]. Probability values less than 0.05 were defined as statistically significant. Statistical analyses were conducted using the statistical package IBM SPSS Statistics version 28.

### Ethics

The LAC-project was approved by the Swedish Ethical Review Authority (Dnr 2020–02979) and follows the principles of the declaration of Helsinki. All participants gave their informed (digital) consent by answering the first survey in the LAC-project.

## Results

Fifteen persons were included in the study, all were women with a mean age of 48 years. The most frequently reported PCC symptoms among the participants were tiredness, breathing difficulties and altered smell/taste. Almost a third had participated in rehabilitation in relation to COVID-19. Sociodemographic characteristics are shown in Table 1. All participants had a total score on Fatigue Severity Scale (FSS) exceeding 4 (i.e., indicating self-reported fatigue) [17].

**Table 1:** Participants’ sociodemographic characteristics and symptoms related to post COVID-19 condition

	Participants (*n* = 15)
**Age (years)**, mean (SD)	48 (6)
**Sex**, (n)	
Female	15
**Married or cohabiting**, (n)	
Yes	14
**Postsecondary education**, (n)	
Yes	12
**Source of income**, (n)	
Employment	8
Sickness benefit	6
Other sources of income	1
**Comorbidities**, (n)	
Yes	8
**Needed hospital care due to COVID-19**,** (**n)	
Yes	2
**Duration of symptoms due to COVID-19 (months)**, mean (SD)	14.6 (2.8)
**Prevalence of common PCC symptoms**, (n)	
Tiredness	14
Breathing difficulties	7
Loss or changed smell/taste	6
Chest pain	5
Joint and muscle pain	4
Coughing	4
**Participated in rehabilitation in relation to COVID-19**, (n)	
Yes	4

**SD** Standard deviation; **n** numbers; **PCC** post COVID-19 condition

At assessment occasion 1, participants spent on average 8 h in SED, 4.3 h in LPA and 23 min in MVPA per day. Results based on accelerometer-data and self-reported outcomes are presented in Table 2.

**Table 2:** Accelerometer-data (min/day) from measurement occasion 1 and self-reported outcomes from questionnaire (*n* = 15)

**Minutes/day**	**Median (IQR)**
SED	476 (383–570)
LPA	260 (204–353)
MVPA	23 (11–64)
**Self-reported outcomes**	**Median (IQR)**
Physical capacity, RPC	5 (4–6)
HRQoL, EQ VAS	50 (30–65)
Fatigue, FSS	6.3 (5.7–6.8)

**SED** Sedentary behaviour; **LPA** Light physical activity; **MVPA** Moderate/vigorous physical activity; **RPC** Rating of Perceived Capacity; **EQ-VAS** EuroQoL-visual analogue scale; **FSS** Fatigue Severity Scale

There was a significant positive correlation (r_s_=0.65) between self-reported physical capacity and MVPA and a significant negative correlation (r_s_ = -0.56) between self-reported fatigue and MVPA but not with SED or LPA. No significant correlations were found between HRQoL and SED, LPA or MVPA (Table 3).

**Table 3:** Correlations (Spearman’s Rho) between self-reported outcomes (physical capacity, HRQoL and fatigue) and median daily time in sedentary behaviour, light, and moderate/vigorous physical activity level (measurement 1)

Self-reported measures		SED (min/day)	LPA (min/day)	MVPA (min/day)
Physical capacity, RPC (*n* = 14)	r_s_	-0.22	0.48	0.65
	p	0.44	0.09	0.01
HRQoL, EQ VAS (*n* = 15)	r_s_	0.21	0.24	0.32
	p	0.45	0.40	0.25
Fatigue, FSS (*n* = 15)	r_s_	-0.32	-0.28	-0.56
	p	0.25	0.32	0.03

**SED** Sedentary behaviour; **LPA** Light physical activity; **MVPA** Moderate/vigorous physical activity; **RPC** Rating of Perceived Capacity; **EQ-VAS** EuroQoL-visual analogue scale; **FSS** Fatigue Severity Scale

Of the 15 participants, 11 had accelerometer data from both assessment occasions and were included in the analyses regarding changes in SED and PA. At the two assessment occasions, the participants spent on average 8.1 respectively 8.6 h per day in SED, 4.7 and 4.3 h in LPA. Although there was a tendency towards more SED and less PA time at assessment occasion 2 compared to assessment occasion 1, the differences were small and non-significant (Table 2 in the supplementary material).

## Discussion

In this study, daily time in MVPA was positively associated with self-reported physical capacity and negatively associated with fatigue but not with HRQoL. SED and LPA were not significantly associated with the self-reported outcomes. Daily SED and PA levels, LPA and MVPA, did not significantly change over a three-month period, according to the accelerometer data.

At measurement occasion 1, the participants spent a median of 23 min in MVPA, with a IQR of 11–64. This means that there were individuals that did not reach the recommended levels of daily PA and therefore may be at risk of other diseases such as cardiovascular disease [18].

There are a few published RCTs showing a beneficial effect of physical training on (among others) fatigue, physical capacity, and quality of life in people with PCC but the level of evidence is low [19]. In our study, physical capacity and fatigue were moderately associated with time spent in MVPA. Higher physical capacity was associated with more daily minutes in MVPA and contrariwise. Our results shows that lower level of self-reported fatigue, more time spent in MVPA, and higher level of physical capacity are associated. However, since we do not know in what direction the associations are, it could also show that higher level of self-reported fatigue, less time spent in MVPA and lower level of physical capacity are associated. This is in line with results from previous research on healthy adults [20] and individuals with chronic obstructive pulmonary disease [21]. Our results, that higher levels of self-reported fatigue were associated with less time in MVPA and contrariwise, correspond well with the findings from a study on participants with multiple sclerosis, where fatigue also is a common symptom (51).

That significant associations were found between physical capacity and time in MVPA but not time in LPA or SED is not surprising since MVPA requires a higher physical capacity than LPA and SED. No change i SED, LPA and MVPA were seen between the two measurement occasions although SED was slightly lower, and PA was slightly higher at the first measurement occasion compared to the second. The accelerometer data were collected in the summer/early autumn when weather conditions are relatively stable. However, the first accelerometer-measurement took place in a period when many Swedes are on vacation, and thus have time for PA, which might be one explanation for the slightly higher PA-levels at this measurement occasion.

Engaging in MVPA on a regular basis is important to maintain good health and reduce the risks of several common diseases. However, in PCC as well as in other conditions, there may be several barriers that could affect engagement in PA. Physical capacity and fatigue could potentially constitute both a barrier and a consequence of engagement in MVPA, but more research is needed to explore the causality of this relationship.

### Limitations

The recruitment process in the LAC-project, through social media, and the inclusion/exclusion criteria for the present study might have induced selection bias and difficulties to generalise the results in the overall PCC population. Social media might favour the recruitment of women [22], and in this study, only women were included in the final analyses which reduces the generalisability of the results. A higher proportion had sickness benefit in this sub-sample compared to all participants in the LAC-project. Furthermore, excluding potential participants that had other diseases such as fibromyalgia and heart condition might have affected the results. One could speculate that persons with comorbidities might be less engaged in PA but the associations between PA and self-reported outcomes would probably not be affected. The number of participants that agreed to wear the accelerometer were modest to begin with and due to a relatively high proportion of missing data, the final sample size was small. Furthermore, four participants lacked valid accelerometer-data from the second measurement which resulted in fewer participants in the analyses responding to the second aim. Furthermore, there is no golden standard of measuring free-living PA and although actigraph is the most used accelerometer, previous research has shown a large variability in measurement properties [23].

Also, the measurement period of three months is relatively short considering that the participants had lived with PCC for more than a year. The natural trajectory for most people with PCC is that a slow reduction of symptoms takes place over time which may explain the non-significant changes in PA between the two measurement occasions [24].

The use of an accelerometer is usually considered a favourable method of measuring PA compared to self-reported PA since the latter might be influenced by different types of bias [25]. By modifying cut points for the minimal accelerometer wear time, as indicated by the sensitivity analysis, effort was taken to ensure unnecessary exclusion of participants.

Despite these limitations, we consider our results valuable as they indicate that people with PCC might be insufficiently physically active and that this might be related to fatigue and low perceived physical capacity. Hence, when planning rehabilitation programs for this group of people, we suggest that PA needs to be introduced at a low level and stepwise increased, not to worsening fatigue. However, more research about how to optimize rehabilitation for this group of people is needed.

## Conclusion

In this study, we found that good perceived physical capacity and low self-reported fatigue were moderately associated with more daily time in MVPA in persons with PCC. Hence, it is important to evaluate physical activity and fatigue when recommending PA among people with PCC.

## Electronic supplementary material

Below is the link to the electronic supplementary material.

Supplementary Material 1

## Data Availability

Research data can be available upon request to the corresponding author.
