# Supplementary material for: Sedentary behaviour and level of physical activity among people with post COVID-19 condition: associated factors and changes over time
Source: BMC Res Notes. 2025 Mar 20;18:119. doi: 10.1186/s13104-025-07177-4 (PMC11927328; doi:10.1186/s13104-025-07177-4)
Supplement: Supplementary file 1 — Supplementary Material 1 [file 13104_2025_7177_MOESM1_ESM.docx]

| **Appendix**  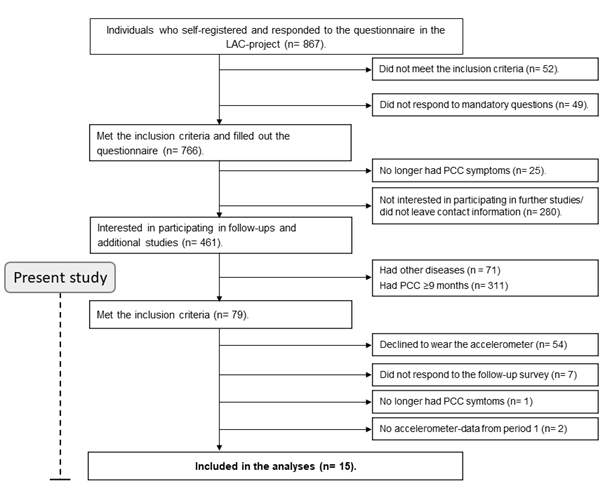  Figure 1. Flowchart of the recruitment process.  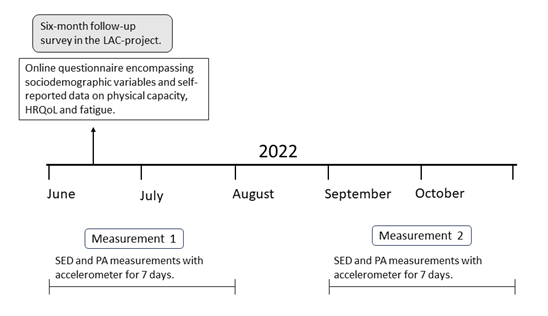  Figure 2. Data collection timeline. |  |  |  |  |
| --- | --- | --- | --- | --- |

| **Table 1.**  Daily time in sedentary behaviour, light physical activity and moderate and/or vigorous activity at the two measurement occasions using different cut points for wear time. Suggested cut points for counts in different intensities by Freedman (2011) was applied. | | | | |
| --- | --- | --- | --- | --- |
| **Measurement occasion and minimum wear time** | **Included (n)** | **SED min/day** Median (IQR) | **LPA min/day** Median (IQR) | **MVPA min/day** Median (IQR) |
| *Measurement 1* |  |  |  |  |
| 4 days, 600 min | N= 13 | 486 (433-572) | 270 (233-354) | 23 (9-66) |
| 4 days, 500 min | N= 15 | 476 (383-571) | 260 (227-353) | 23 (11-64) |
| 3 days, 600 min | N= 14 | 481 (413-571) | 265 (235-353) | 23 (10-65) |
| 3 days, 500 min | N= 15 | 476 (383-571) | 260 (204-353) | 23 (11-64) |
| *Measurement 2* |  |  |  |  |
| 4 days 600 min | N= 8 | 507 (457-573) | 255 (226-294) | 28 (14-46) |
| 4 days 500 min | N= 10 | 501 (423-566) | 251 (212-289) | 18 (13-42) |
| 3 days, 600 min | N= 10 | 526 (478-577) | 255 (222-289) | 26 (11-42) |
| 3 days, 500 min | N= 11 | 514 (425-576) | 259 (214-286) | 21 (14-41) |
| **SED** Sedentary behaviour; **LPA** Light physical activity; **MVPA** moderate and/or vigorous physical activity; **IQR** Interquartile range   \| **Table 2.** \| \| \| \| \| --- \| --- \| --- \| --- \| \| Differences between measurement occasions in median (IQR) daily minutes in sedentary behaviour, light, and moderate/vigorous physical activity. Only participants with data from both measurements were included (n =11). \| \| \| \| \|  \| **Measurement 1** \| **Measurement 2** \| **Sig (2-tailed)** \| \| Minutes/day \| Median (IQR) \| Median (IQR) \| P-value \| \| SED \| 486 (370-571) \| 514 (425-576) \| 0.11 \| \| LPA \| 280 (204-354) \| 259 (214-286) \| 0.06 \| \| MVPA \| 23 (13-64) \| 21 (14-41) \| 0.306 \| \| **IQR** Interquartile range; **SED** Sedentary behaviour; **LPA** Light physical activity; **MVPA** moderate or/and vigorous physical activity  Wilcoxon signed rank test was used to analyse the data. \| \| \| \| | | | | |
